# Dendritic Morphology of Hippocampal and Amygdalar Neurons in Adolescent Mice Is Resilient to Genetic Differences in Stress Reactivity

**DOI:** 10.1371/journal.pone.0038971

**Published:** 2012-06-12

**Authors:** Anup G. Pillai, Danielle de Jong, Sofia Kanatsou, Harm Krugers, Alana Knapman, Jan-Michael Heinzmann, Florian Holsboer, Rainer Landgraf, Marian Joëls, Chadi Touma

**Affiliations:** 1 Department of Neuroscience and Pharmacology, RMI, University Medical Center, Utrecht, The Netherlands; 2 Max Planck Institute of Psychiatry, Munich, Germany; 3 Swammerdam Institute for Life Sciences, Center for Neurosciences, University of Amsterdam, The Netherlands; Institut National de la Santé et de la Recherche Médicale, France

## Abstract

Many studies have shown that chronic stress or corticosterone over-exposure in rodents leads to extensive dendritic remodeling, particularly of principal neurons in the CA3 hippocampal area and the basolateral amygdala. We here investigated to what extent genetic predisposition of mice to high versus low stress reactivity, achieved through selective breeding of CD-1 mice, is also associated with structural plasticity in Golgi-stained neurons. Earlier, it was shown that the highly stress reactive (HR) compared to the intermediate (IR) and low (LR) stress reactive mice line presents a phenotype, with respect to neuroendocrine parameters, sleep architecture, emotional behavior and cognition, that recapitulates some of the features observed in patients suffering from major depression. In late adolescent males of the HR, IR, and LR mouse lines, we observed no significant differences in total dendritic length, number of branch points and branch tips, summated tip order, number of primary dendrites or dendritic complexity of either CA3 pyramidal neurons (apical as well as basal dendrites) or principal neurons in the basolateral amygdala. Apical dendrites of CA1 pyramidal neurons were also unaffected by the differences in stress reactivity of the animals; marginally higher length and complexity of the basal dendrites were found in LR compared to IR but not HR mice. In the same CA1 pyramidal neurons, spine density of distal apical tertiary dendrites was significantly higher in LR compared to IR or HR animals. We tentatively conclude that the dendritic complexity of principal hippocampal and amygdala neurons is remarkably stable in the light of a genetic predisposition to high versus low stress reactivity, while spine density seems more plastic. The latter possibly contributes to the behavioral phenotype of LR versus HR animals.

## Introduction

Altered reactivity and feedback regulation of the hypothalamo-pituitary-adrenal (HPA) axis in response to stressors are considered to be risk factors for the precipitation of psychiatric disorders, including major depression [1], [2]. For instance, high-risk probands of major depression already show increased HPA axis reactivity prior to the manifestation of any clinical symptom [3]–[6], suggesting that altered HPA axis reactivity may actually represent a predisposing trait. Moreover, elevated corticosteroid levels, particularly during the circadian trough [7]–[9], as well as impaired responsiveness of the HPA axis to negative feedback by glucocorticoids have been reported in the majority of depressives [10], [11]. HPA axis function in patients suffering from depression is partly normalized upon treatment; the degree of normalization predicts relapse probability [12]–[17]. Recent structural and functional neuroimaging approaches have revealed consistent though small abnormalities in the brains of depressives [18]–[22], although moderating factors such as gender and early life adversity play an important role [23]–[26].

To study the putative neuronal mechanism underlying changes in brain structure and function associated with altered HPA axis reactivity, several animal models are available. One of these models involves mouse lines selectively bred for differences in their corticosterone response to a moderate psychological stressor [27], eventually resulting in a distinct phenotype characterized by high (HR), intermediate (IR) or low (LR) reactivity of the HPA axis to acute stressors. HR compared to LR mice display a hyper-responsive adrenal gland, adrenal hypertrophy, reduced bodyweight and elevated levels of glucocorticoids during the circadian trough. Furthermore, HR mice show changes in sleep architecture, hyperactive coping behavior and cognitive deficits in several behavioral paradigms, i.e. a phenotype that recapitulates some features observed in depressive illness [28]–[31].

Rodents exposed to chronic stress or very high levels of corticosteroids in (young) adulthood consistently show reduced dendritic complexity of hippocampal CA3 pyramidal neurons [32]–[34]; CA1 neurons are far less affected [35]–[37]. By contrast, principal neurons in the basolateral amygdala appear more complex after chronic stress [38]–[40]. It has been suggested that this structural plasticity may contribute to the (small) limbic volume changes associated with major depression [41]–[44]. While chronic stress in adulthood captures aspects (mostly the environmental influence) of the risk on depression, it lacks the element of genetic predisposition. To specifically address this element, we used the HR, IR and LR mouse lines to examine if their genetic predisposition to differences in stress reactivity also yields structural changes –i.e. altered dendritic morphology- in principal neurons of the hippocampal CA1 and CA3 areas and in the basolateral amygdala.

## Materials and Methods

### Experimental subjects

All animals used in this study were male mice from the 12^th^ breeding generation of the stress reactivity mouse model (for details see [27]). Using a selective breeding approach, the three mouse lines (HR, IR, and LR) were generated from CD-1 mice (Charles River Laboratories, Sulzfeld, Germany). The animals were tested for HPA axis reactivity at the age of about five weeks (see below) and killed two weeks later.

All animals, after weaning (at the age of about 4 weeks), were housed in groups of two to four mice in transparent polycarbonate cages (standard Macrolon cages type III, 38×22×15 cm3) with wood chips as bedding and wood shavings as nesting material (Product codes: LTE E-001 and NBF E-011, ABEDD – LAB and VET Service GmbH, Vienna, Austria). The animal housing rooms as well as the experimental rooms were maintained under standard laboratory conditions (light–dark cycle: 12∶12 h, lights on at 08:00 h; temperature: 22±1°C; relative humidity: 55±10%). Commercial mouse diet (Altromin no. 1324, Altromin GmbH, Lage, Germany) and bottled tap water were available ad libitum. All efforts were made to reduce suffering and discomfort to the animals at all experimental stages.

The presented work complies with current regulations covering animal experimentation in Germany and the EU (European Communities Council Directive 86/609/EEC). All experiments were announced to the appropriate local authority and were approved by the ‘Animal Welfare Officer’ of the Max Planck Institute of Psychiatry (Az. 55.2-1-54-2531-64-07).

### Power analysis

A power analysis was conducted *a priori* to determine the sample size for the study, as mandated by the current regulations for animal experimentation in the EU (Communities Council Directive 86/609/EEC). Since a pilot study was not possible, we used values from published reports to calculate the sample size. A survey of literature on stress effects in the limbic region showed that the effects of chronic stress are most evident in the hippocampal CA3 region [32]–[35], [38]. We therefore computed the effect size from a study that investigated the effects of chronic stress on dendritic morphology of CA3 hippocampal neurons [38]. Mean values of total apical dendritic length of CA3 neurons from three groups (control: 2113 µm, predictable stress: 1498 µm, unpredictable stress: 1749 µm) with an averaged standard deviation (424.3) and sample size per/group (50 neurons) provided an effect size of 0.6. Half of the effect size for chronic stress (i.e. 0.3) was taken as a conservative estimate for the effect size of our study, considering the milder experimental conditions. With an effect size of 0.3, an alpha error probability of 0.05 and a power of 0.9 (and using a one-way ANOVA comparing three experimental groups), we calculated a required sample size of in total 144 neurons in the CA3 area.

Establishing the number of animals in which these 144 cells had to be collected involves a trade-off between the variation within animals and between animals. In our approach we put emphasis on the latter. By aiming for 8 animals per experimental group and 3 experimental groups (N = 24 animals in total), we required on average 6 neurons per animal to reach n = 144 cells; this was liberally interpreted as 4–8 cells per animal. In reality, we included 188 CA3 neurons in our overall analysis. The values we determined for the number of animals per group and cells per animal were very similar to the ones used by another study addressing the effects of stress on CA3 morphology (6–8 CA3 neurons per animal, 5–6 animals per group; see ref. [32]).

### Stress reactivity test (SRT) and corticosterone measurement

The SRT was used to test the HPA axis reactivity of each animal (n = 24 in total) and consisted of a brief period of restraint stress (15 min) in a 50 ml plastic tube (for details see [27]). The tube is perforated for ventilation and has an opening at the rear end for the tail. Immediately before and after exposure to the stressor, a small incision was made in the tail, and blood samples were collected from the ventral tail vessel using heparin coated capillaries (for details see [27]). All blood sampling and restraint were performed in a separate room adjacent to the animal housing room, and within a maximum time of 2 min from disturbing the animal's cage (all mice in a cage were treated in parallel). The blood was quickly centrifuged at high speed (14,800 g) for 5 min to separate the plasma and was kept frozen (−20°C) until the corticosterone assay was carried out. In order to avoid any influence of fluctuating baseline corticosterone levels during circadian variations, the SRT was always performed between 0900-1100 h when circulating corticosterone levels are low [29], [45]–[47]. Plasma corticosterone concentration was measured using RIA kits (MP Biomedicals, Solon, Ohio, USA). Intra- and inter-assay coefficients of variation were both below 10%.

### Staining and preparation of histological sections

At the age of seven weeks, i.e. two weeks after SRT testing, the animals were sacrificed by decapitation under isoflurane anesthesia. The brains were quickly dissected and immediately immersed in the Golgi-Cox fixative consisting of the following components: 5% potassiumbichromate, 5% mercurichloride, 5% potassiumchromate and Milli-Q water mixed at a ratio of 5∶5∶4∶10. After 5 days, the brains, kept in dark bottles that contained the fixative, were replenished with a new stock. Complete impregnation was obtained after 21–28 days in the Golgi-Cox solution.

Embedding of the brains involved a nine days' procedure. On day 1, brains were thoroughly rinsed with water followed by overnight dehydration in 70% ethanol. On day 2, brains were transferred to 96% ethanol and allowed to remain there overnight. On the next day, the brains, after immersion in absolute ethanol for 8 h, were subsequently moved to a beaker containing a mixture of ethanol and ether (1∶1) and left undisturbed for 16 h. For embedding, the brains were kept in 3% and 6% celloidine overnight on day 4 and 5, respectively. For the next two days, they remained in 12% celloidine. On day 8, the brains were transferred to paper boxes small enough to contain the brain and were filled with 12% celloidine. The boxes were gently stacked in a beaker and filled with chloroform. The procedure required the celloidine embedded brains to be in chloroform for 16 h. Lastly, the brains were shifted to bottles containing 70% ethanol and kept refrigerated (4°C) until sectioning and staining.

Coronal sections (200 µm) of the brain were cut on a vibrating slicer (Vibratome, USA) and were stored (±24 hrs) in 70% ethanol until staining. For the staining, the sections were first treated for 30 minutes in ammonia solution (16%) and were subsequently fixed using sodium thiosulphate (1%). Between every step the sections were rinsed with water for 2–5 min and were kept in ethanol (70%)-containing petridishes, before clearing with concentrated ethanol (96%) and butanol. The sections, cleared twice in histoclear for 5 min each, were then cover-slipped with enthalan and dried in a fume hood for 2–3 days.

### Morphological analysis

For morphological quantification of hippocampal neurons, pyramidal shaped neurons were selected from both the CA1 and CA3 region of the dorsal hippocampus (Bregma −2.6 to −3.8 mm). Dendritic tracing in the amygdala was restricted to pyramidal and stellate shaped neurons from the basolateral region (Bregma −0.58 to −3.16 mm) of the amygdala. On average, 4–8 neurons from both hemispheres were included per animal, for each brain region. Before tracing, neurons were visually inspected for the integrity of dendritic branches and were discarded if any of the proximal dendritic branches were cut. Neurons with dendrites running deeper into the section or clustered with other stained cells were also not included in the study due to difficulties in tracing them accurately. Image stacks (60–130; 1 µm thick) of the neurons that met the above criterions, were recorded with Image-Pro Plus, (Media Cybernetics, Sliver Spring, MD, USA), using an Axioplan 2 (Zeiss, Germany) microscope, equipped with a CCD camera (Media Cybernetics), at 40× objective. All sections were coded and the experimenter was unaware of the groups until the analysis was completed.

Neuron tracing was performed using the drawing tool Neurodraw (developed at and generously supplied by the Netherlands Institute for Neurosciences, Amsterdam, The Netherlands) in combination with Image-Pro Plus. Sholl analysis was later carried out in L-Measure (http://cng.gmu.edu:8080/Lm/). Additionally, we also used Neuromantic (http://www.reading.ac.uk/neuromantic/) and NeuronStudio (http://research.mssm.edu/cnic/tools-ns.html) for some of the analysis and 3D visualization of neuron traces. Dendritic complexity index (DCI) was determined from the following equation, DCI = (Σ branch tip orders + # branch tips)×(total dendritic length/total number of primary dendrites). Branch tip order, an integer value assigned to each terminal tip of the neuron's dendrite, equals the number of sister branches emanating from the dendritic segment between a particular terminal tip and cell body.

### Spine density measurements

Spines were quantified from images acquired through a Zeiss Axiosope microscope (63× plan apochromat oil immersion objective, 1.4 NA) using the same software as described previously (Neurodraw in combination with Image-Pro Plus). All protrusions, regardless of their morphological features (thin vs. stubby, with or without terminal bulbous expansions) that were in direct continuity with the parent dendritic shaft were counted as spines. For the purpose of this study, we restricted our analysis to the spines on the tertiary apical dendritic branches of CA1 pyramidal neurons, located at 120 to 300 µm from the soma.

The following schema was employed to deduce the order of a particular dendritic segment. The main apical shaft (easily identifiable by its thickness) was always considered as the primary branch whose daughter dendrites form the secondary branches which further bifurcate to tertiary dendritic segments. On average for each neuron (4–6 per animal), three distal apical tertiary branches (length: 15 µm; diameter ∼1.0 to 1.5 µm) fulfilling the following criteria were randomly chosen: (1) complete staining of both the parent dendritic shaft and all the visible spines; (2) dendritic segments had to be straight and remain in a single focal-plane, ensuring that a 2D projection length of the dendrite approximated to its actual 3D length; (3) the first 15 µm of the tertiary dendritic segment was within the target distance (120–300 µm) from the soma. In nearly all cases, the three segments were located on different branches, but in a few instances we analyzed two segments from the same dendritic branch. Only spines that were clearly distinguishable and not emanating from either directly above or below the dendritic shaft were counted. Therefore, the spines/15 µm values reported in this study underestimate the actual spine density of CA1 pyramidal neurons. We did not attempt to correct for these discrepancies, because the use of visible spine counts for comparison between experimental conditions, as per the strict criteria we employed, had been validated previously [48]. Mean spine density (per 15 µm) was obtained by averaging the values per neuron (∼3 dendritic segments/neuron). The spine density analysis was performed by an experimenter blind to the experimental groups.

### Statistical analysis

All data was normally distributed (Kolmogorov-Smirnov test) and hence was analyzed using parametric tests. One-way ANOVA followed by *post hoc* (Bonferroni) testing was used to compute statistical significance between groups in experiments that involved more than two groups. Data that did not comply with variance homogeneity (Levene's test) were additionally analyzed using Welch's test. Statistical significances for segmental dendritic plots (Sholl analysis) were obtained from mixed factors (within and between subject factors) repeated measures ANOVA with adequate corrections (Greenhouse-Geisser) on the significant values wherever the sphericity assumption was not met. Differences were considered significant if their probability of occurring by chance was less than 5% (p<0.05). Statistical analyses were performed using either PSAW Statistics 18 or R (www.r-project.org).

For each parameter and each experimental group, we determined the mean value by averaging the observations of all cells per experimental group. This is an approach often used in studies on changes in dendritic complexity after stress (see e.g. ref. [38]–[40]). These data are shown in the figures and tables. However, one can argue that characteristics of the various cells per animal are not truly independent measures. Therefore, we also report (in Tables 1 and 2) the ANOVA *p*-values based on the averages of all *animals* per group.

**Table 1:** Morphological analysis of apical and basal dendrites in CA1 and CA3 hippocampal cells from HR, IR and LR animals.

CA3 region of the hippocampus								
	Apical			Basal				
Groups	HR	IR	LR	HR	IR	LR	F-value/*p*-value analysis by cells (animals)	
N (animals), n (cells)	8, 71	8, 57	8, 60	8, 71	8, 57	8, 60	Apical	Basal
**Total dendritic length (µm)**	1117±52	1069±63	1007±59	897±50	754±43	859±58	0.9/**0.4** (0.4/**0.7**)	2.1/**0.1** (2.0/**0.2**)
**Total # branch points**	10.6±0.5	10.4±0.7	9.5±0.6	9.1±0.5	7.8±0.5	8.6±0.6	0.9/**0.4** (0.6/**0.6**)	1.4/**0.2** (2.0/**0.2**)
**Total # branch tips**	11.8±0.5	11.6±0.7	10.7±0.6	12.5±0.6	11.1±0.5	12.0±0.6	0.9/**0.4** (0.5/**0.6**)	1.4/**0.2** (2.0/**0.2**)
**Total tip order**	48.2±2.9	49.2±4.6	42.5±3.8	30.6±2.2	25.3±2.0	28.5±2.4	0.9/**0.4** (0.6/**0.6**)	1.4/**0.3** (2.2/**0.1**)
**Total # primary dendrites**	1.2±0.06	1.2±0.06	1.2±0.07	3.4±0.14	3.3±0.14	3.4±0.18	0.06/**0.9** (0.6/**0.6**)	0.14/**0.9** (0.6/**0.6**)
**Dendritic complexity**	68495±6972	68515±9960	58710±7856	14360±1677	10365±1401	13389±2333	0.5/**0.6** (0.5/**0.6**)	1.2/**0.3** (2.3/**0.1**)

CA1 region of the hippocampus								
	Apical			Basal				
Groups	HR	IR	LR	HR	IR	LR	F-value/*p*-value analysis by cells (animals)	
N (animals), n (cells)	8, 34	8, 37	7, 27	8, 34	8, 37	7, 27	Apical	Basal
**Total dendritic length (µm)**	869±57	902±72	896±66	692±51	583±47	742±68	0.07/**0.9** (0.05/**1.0**)	2.3/**0.1** (3.9/**0.04** *)
**Total # branch points**	11.1±1.0	12.0±1.1	12.5±1.3	7.8±0.8	6.9±0.6	9.0±0.9	0.4/**0.7** (0.3/**0.7**)	2.0/**0.1** (2.2/**0.1**)
**Total # branch tips**	12.1±1.0	13.2±1.2	13.6±1.3	11.6±0.7	10.6±0.8	12.6±0.9	0.4/**0.7** (0.3/**0.7**)	1.4/**0.3** (1.9/**0.2**)
**Total tip order**	63.6±7.5	70.1±9.3	75.4±11.0	26.6±3.4	21.2±2.2	29.6±3.3	0.4/**0.7** (0.4/**0.7**)	2.1/**0.1** (2.1/**0.2**)
**Total # primary dendrites**	1.1±0.04	1.2±0.07	1.1±0.09	3.8±0.2	3.7±0.3	3.6±0.2	1.1/**0.3** (1.4/**0.3**)	0.22/**0.8** (0.3/**0.8**)
**Dendritic complexity**	74980±11398	79977±15016	85359±15671	10007±1795	6065±821	10796±1725	0.12/**0.9** (0.13/**0.9**)	3.1/**0.049** # (3.5/**0.049** #)

Per experimental group and parameter, we determined the mean±SEM by averaging the values per cell and these were compared with ANOVA (right column). In addition to this, we also report comparisons (ANOVA) based on the averages of all *animals* per group (F/***p***-value in parentheses). *Post hoc*: adj. Bonferroni,

* *p*<0.05; 0.05>.

# *p*<0.1.

**Table 2:** Morphological analysis of dendrites of basolateral amygdala principal neurons from HR, IR and LR animals.

Basolateral amygdala				
Groups	HR	IR	LR	
N (animals), n (cells)	6, 35	7, 32	5, 19	F-value/*p*-value analysis by cells (animals)
**Total dendritic length (µm)**	1618±79	1694±97	1544±111	0.6**/0.6** (0.02/**1.0**)
**Total # branch points**	15.0±0.8	15.0±1.0	15.9±1.2	0.2/**0.8** (0.3/**0.8**)
**Total # branch tips**	20.8±1.0	20.9±1.2	22.1±1.3	0.3/**0.7** (0.2/**0.8**)
**Total tip order**	50.0±3.5	47.8±3.5	51.5±4.9	0.2/**0.8** (0.4/**0.7**)
**Total # primary dendrites**	5.7±0.2	5.9±0.3	6.2±0.3	0.4/**0.7** (0.06/**0.9**)
**Dendritic complexity**	21507±1988	21521±1927	20768±2965	0.03/**1.0** (0.3/**0.8**)

Per experimental group and parameter, we determined the mean±SEM by averaging the values obtained for all cells per group. The averages for the three experimental groups were compared with ANOVA (the *p*-values on the right). In addition to this, we also compared the groups based on the averages of all *animals* per group (ANOVA, F/***p***-value in parentheses on the right).

## Results

### Stress-induced plasma corticosterone concentrations are different between HR, IR and LR mice

**Figure 1:**
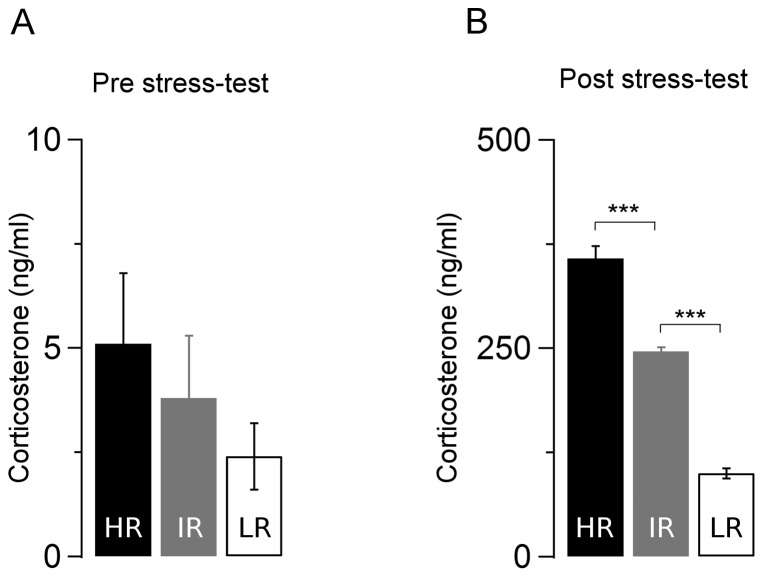
Corticosterone levels in high, intermediate and low stress reactive mice after exposure to a brief stressor. ***A & B*** **,** averaged values of plasma corticosterone concentration measured immediately before (pre stress-test: HR: 5±1.7, IR: 4±1.4, LR: 2.4±0.8) after the SRT (post stress-test: HR: 358±15, IR: 246±5, LR: 100±6) in mice genetically selected for differences in stress reactivity (N, HR: 8, IR: 8, LR: 8). ***p<0.001 Bonferroni adj. *post-hoc* test. Values in ng/ml (mean±sem).

Basal corticosterone concentrations in blood samples were collected from mice under unstressed conditions (immediately before the SRT). Although on average LR animals displayed 2-fold lower plasma corticosterone levels than did HR mice, the levels of the three groups did not differ significantly (N = 8 animals/group, F_(2,21)_ = 1, *p*>0.3; ANOVA, Fig. 1A). A single episode of 15-min restraint stress caused a significant increase in plasma corticosterone in all groups when compared to the baseline levels (N = 8 animals/group, t_(23)_ = −10.2, *p*<0.001; paired T-test, Fig. 1B). Moreover, HR, IR and LR animals displayed sequentially increasing concentrations of corticosterone in response to the SRT (F_(2,21)_ = 178.4, *p*<0.001, ANOVA). Accordingly, the HR group exhibited the highest levels of corticosterone (HR vs LR: *p*<0.001; Bonferroni *post hoc*), followed by the IR mice whose values were between HR (*p*<0.001) and the LR mice (*p*<0.001) which had the lowest corticosterone concentration. These results, which are in agreement with previous reports on the stress reactivity mouse model [27], provided a robust neuroendocrinological phenotype to test the consequences of enduring abnormal HPA axis reactivity on cellular plasticity of limbic neurons in late adolescent mice.

### Dendritic architecture of hippocampal CA3 pyramidal neurons did not differ between HR, IR and LR mice

**Figure 2:**
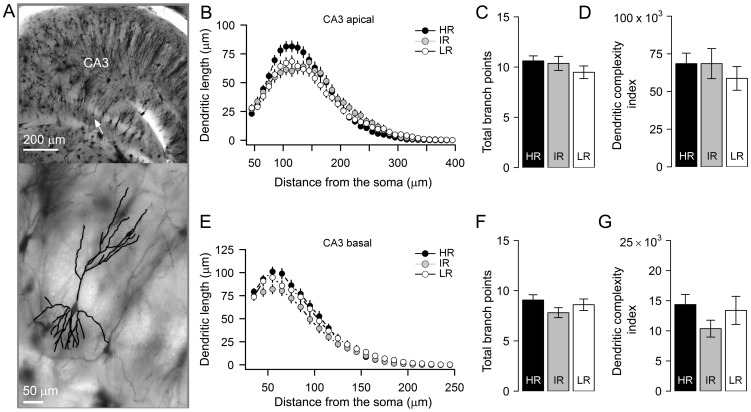
Hippocampal CA3 pyramidal neurons from high, low and intermediate reactive mice exhibit similar dendritic morphology. ***A*** **,** ***top***, coronal photomicrograph of the CA3 region from a Golgi-stained brain section. The white arrow indicates the position of the representative neuron shown at higher magnification below. ***A, bottom***, dendritic trace of a typical CA3 pyramidal neuron is superimposed on its image. ***B & E***, Sholl plots indicate the distribution of apical and basal dendritic length at increasing distance from the center of the cell body. ***C, D, F, & G***, average values of total dendritic bifurcations (mean±sem, n, *apical*, HR: 11±1, 71; IR: 10±1, 57; LR: 10±1, 60; *basal*: HR: 9±1; IR: 8±1; LR: 9±1) and complexity (mean±sem, *apical*, HR: 68495±6972; IR: 68515±9960; LR: 58710±7856; *basal*, HR: 14360±1677; IR: 10365±1401; LR: 13387±2333) are similar in neurons from HR, IR, and LR mice.

An increasing number of reports indicates that the apical dendrites of CA3 pyramidal neurons in particular undergo severe dendritic remodeling following repeated exposure to either stress or corticosterone treatment [32], [34], [38]. We, therefore, initially focused on the effects of altered stress reactivity on apical and basal dendritic structure of hippocampal CA3 pyramidal neurons in HR, IR and LR mice (Fig. 2A). Between the three groups no difference was found in total apical dendritic length, the number of branch points and branch tips, summated tip order or number of primary dendrites (F_(2,185)_<1, *p*>0.4, One-way ANOVA; see Table 1 for details and Fig. 2C for illustration). In accordance, the dendritic complexity index of apical dendrites –which is calculated from these parameters- was also similar (F_(2,185)_ = 0.5, *p*>0.6, Fig. 2D) between HR, IR and LR mice. To further investigate the effects of altered stress reactivity on cellular morphology, a segmental (Sholl) analysis was performed to examine the changes in dendritic length as a function of radial distance from the cell body (Fig. 2B). We found a significant interaction between variations in stress-reactivity and segmental dendritic length in CA3 apical dendrites (stress reactivity x segmental dendritic length; F_(58,5365)_ = 3, *p*<0.001, two-way mixed ANOVA), indicating that stress reactivity is associated with a different distribution of dendritic branches over the entire tree. However, there was no significant main effect of stress reactivity (F_(2,185)_ = 0.96, *p*>0.3; between groups, mixed ANOVA) precluding post-hoc comparison per segment.

A similar analysis was performed on the basal region of CA3 pyramidal neurons. Mean values of total dendritic length, the total number of primary branches, number of branch tips, the branch tip orders (Table 1), the number of branch points (F_(2,185)_<2, *p*>0.1, Fig. 2F) and dendritic complexity (F_(2,185)_ = 1.2, *p*>0.3, Fig. 2G) did not differ significantly between HR, IR and LR mice. In agreement with the above measures on total basal-dendritic tree, Sholl analysis on segmental dendritic length also revealed a striking similarity between the three groups. This is evident from the absence of any significant main effect (F_(2,185)_ = 2, *p*>0.1, between groups) although an interaction was still present (F_(58,5365)_ = 1.7, *p*<0.001; stress reactivity x segmental dendritic length, two-way mixed factors ANOVA, Fig. 2E).

### Principal neurons of the basolateral amygdala are comparable in late adolescent mice that differ in stress reactivity

In addition to CA3 neurons, principal neurons of the basolateral amygdala undergo substantial dendritic remodeling following various stress paradigms, albeit in the opposite direction [38]–[40], [49]. Specifically, chronic stress leads to dendritic growth of basolateral amygdala neurons. We therefore next analyzed the effect of differences in HPA axis reactivity on BLA principal neurons.

**Figure 3:**
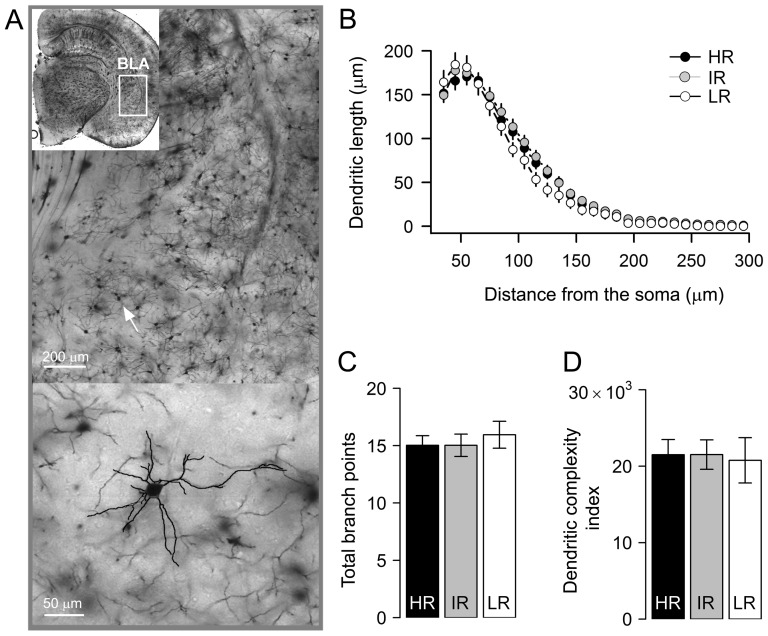
Dendritic morphology of amygdala neurons is not different in high, intermediate and low stress reactivity mice. ***A*** **,** ***top***, representative photomicrograph of a Golgi-stained section containing the basolateral amygdala (***inset***: BLA is indicated by the white square on a hemisected coronal brain section). ***A, bottom***, dendritic tracks of a sample BLA principal neuron (*white arrow*) is superimposed on its image. ***B***, Sholl plots of dendritic length at increasing distance from the centre of the cell body is similar across groups. ***C & D***, values of total basal dendritic bifurcations (mean±sem, n, HR:15±1, 35; IR:15±1, 32; LR:16±1, 19) and complexity (mean±sem, HR:21507±1988; IR:21521±1927; LR:20768±2965), averaged across cells, did not differ between groups.

A total of 86 neurons were traced from the basolateral amygdala of mice from all three groups. Average values of total dendritic length, number of branch points (Fig. 3C) and tips, the summated branch tip orders and the total number of primary branches were not statistically different between HR, IR and LR mice (Table 2). We further observed that mean values of the dendritic complexity index were not at all affected by HPA axis alterations in these mice (F_(2,83)_ = 0.03, *p* = 1.0, Fig. 3D). The results from the Sholl analysis also supported the conclusion that dendritic architecture of BLA neurons is largely unaffected by inherited variations in stress reactivity (Fig. 3B). This is evident from the complete overlap of dendritic length distributions from HR, IR and LR mice (Main effect of stress reactivity: F_(2,83)_ = 0.3, *p*>0.6; stress reactivity x segmental dendritic length: F_(32,1328)_ = 1.4, *p*>0.06, two-way mixed factors ANOVA).

### Morphology of CA1 pyramidal neurons is marginally affected by variations in stress reactivity

**Figure 4:**
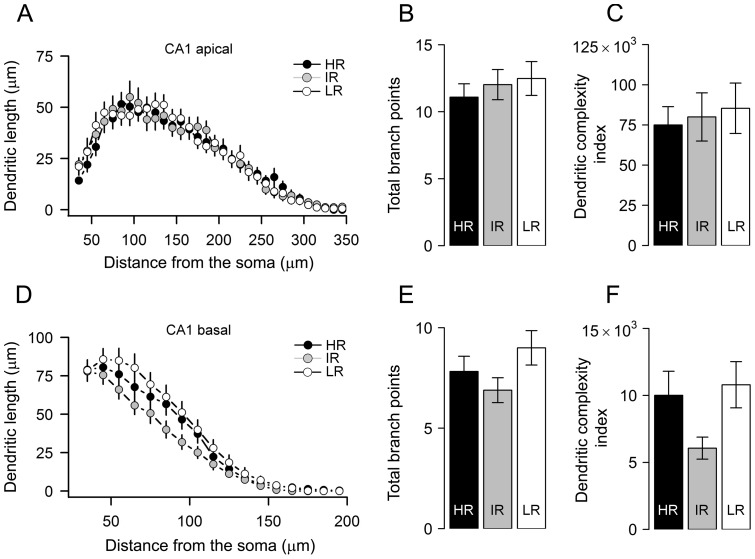
Apical dendrites of CA1 pyramidal neurons are similar between high, intermediate and low stress reactivity mice. ***A & D***, Sholl plots indicate the distribution of dendritic length (apical & basal) at increasing distance from the center of the cell body. ***B, C, E & F***, average values of total dendritic bifurcations (mean±sem, n, *apical*, HR: 11±1, 34; IR: 12±1, 37; LR: 13±1, 27, *basal*: HR: 8±1; IR: 7±1; LR: 9±1) and complexity (mean±sem, *apical*, HR: 74980±11398; IR: 79977±15016; LR: 85358±15671, *basal*, HR: 10007±1795; IR: 6065±822; LR: 10796±1724), averaged across cells, were not statistically different between groups.

Having found no effect of differences in stress reactivity between the three mouse lines on the morphology of CA3 pyramidal neurons and BLA principal cells, we next shifted our focus to the CA1 region of the hippocampus. Similar to CA3 neurons, the apical dendrites of CA1 pyramidal neurons of HR, IR, and LR mice were not significantly different (one-way ANOVA, details in Table 1) in total dendritic length, the total number of primary branches, number of branch tips, the summated branch tip order and the number of branch points (Fig. 4B) as well as dendritic complexity index (Fig. 4C). We further observed that segmental distributions of CA1 apical dendritic lengths from HR, IR and LR mice were also remarkably similar (F_(42,1995)_ = 0.62, *p*>0.9, two-way mixed factors ANOVA, Fig. 4A) across the three mouse lines.

Similar to the observations in apical dendrites, basal dendrites of CA1 neurons from HR, IR and LR animals were generally not significantly different from each other with respect to the parameters measured (Table 1). Neither the total number of branch nodes (Fig. 4E), total dendritic length, the number of primary dendrites and summated tip branch order differed between the three groups (Table 1). While dendritic complexity showed marginal differences in neurons from HR and LR mice compared to IR, (change w.r.t. IR, HR: 65%, LR: 78%; F_(2,95)_ = 3.1, *p* = 0.049, one-way ANOVA), further *post hoc* analyses revealed no significant differences between groups (*p*>0.08, *post hoc*: adj. Bonferroni). However, statistical analysis based on values averaged per animal indicated a significant increase in the total dendritic length of LR animals compared to IR mice (change w.r.t IR, LR: 26%, *p*<0.05, *post hoc*: adj. Bonferroni, see Table 1).

To elaborate further on the marginal increase that we observed on the basal dendritic complexity of CA1 neurons from HR and LR animals, additional Sholl analysis on the entire basal dendrites were carried out for all the three groups (Fig. 4D). Similar to the lack of effect on total CA1 basal dendrites, we did not find a significant effect of HPA axis reactivity on the distribution of basal CA1 dendritic lengths at varying distances from the cell body (F_(26,1235)_ = 1.3, *p*>0.1, two-way mixed factors ANOVA).

### Spine density at the tertiary dendritic branches of hippocampal CA1 pyramidal neurons is significantly higher in LR animals

Results from several studies indicate that dendritic spines are a major site for stress-induced plasticity in the hippocampus [50], [51]. Spine density of CA1 apical dendrites in particular was shown to be reduced after a period of exposure to elevated corticosteroid levels. We therefore hypothesized that variations in stress reactivity might eventually lead to alterations in hippocampal spine number, at least in the CA1 area.

**Figure 5:**
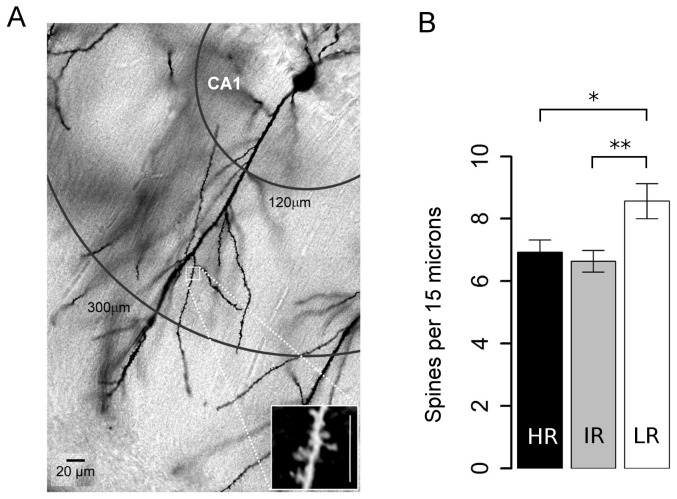
Spine density of hippocampal CA1 pyramidal neurons are different in mice with variations in stress responsivity. ***A***, a schematic illustration of the location of spine density measurements carried out on the tertiary dendritic segments of the stratum radiatum layer of CA1 pyramidal neurons (Sholl distance from the soma: 120–300 µm). ***Inset***, a magnified image of the sampled region (*scale bar*: 15 µm). ***B***, Spine density of the tertiary apical dendritic segments (15 µm) averaged across cells (mean±sem, n, HR: 6.9±0.4, 34; IR: 6.6±0.3, 35; LR: 8.6±0.6, 29) are significantly higher in neurons from LR compared to IR or HR mice. **p<0.01, *p<0.05 Bonferroni adj. *post-hoc* test.

Spines from 282 dendritic segments were analyzed to obtain the averaged spine density of 98 CA1 pyramidal neurons in total. All spine measurements were confined to the tertiary dendritic segments of the CA1 stratum radiatum layer (120–300 µm from the soma) of the hippocampus (see typical examples in Fig. 5A). A significant difference was observed between the three groups (ANOVA: F_(2,95)_ = 5.58, *p* = 0.005). *Post hoc* multiple comparisons of the means revealed that the spine density in cells from LR animals was significantly higher compared to cells from either IR (*p* = 0.007) or HR animals (*p* = 0.028; Fig. 5B). It should be noted, though, that group averages based on animals (∼6 animals per group) instead of cells did not yield significant differences (HR: 7.2±0.86, IR: 6.5±0.34, LR: 8.5±1.0, F_(2,16)_ = 1.7, *p* = 0.22; one-way ANOVA).

## Discussion

Chronic stress in adulthood or long-term over-exposure of rodents to corticosterone is associated with reduced dendritic complexity of CA3 (and to a lesser extent CA1) pyramidal neurons [32]–[35]. Conversely, a remarkable growth of principal neurons in the basolateral amygdala has been observed after chronic restraint stress [36], [38]. It has been suggested that this may bear relevance to the structural changes observed in association with major depression [20], [52]–[54], since aberrant function of the HPA axis is considered to be a potential predisposing trait for the precipitation of depressive symptoms, which may have a genetic origin [3]. Instead of using chronic stress or corticosteroid exposure, mostly reflecting a rather extreme ‘state’ of the animal, we here used genetic mouse lines selectively bred for their stress reactivity, a presumed vulnerability ‘trait’ [27]–[29], [31], [55]. The main conclusion of our present study is that in this model for genetic differences in stress reactivity (high, intermediate or low) the dendritic complexity of principal neurons in the CA3 and CA1 hippocampal areas and in the basolateral amygdala is highly comparable for all three lines; however, spine density, at least in the CA1 area, seems more plastic.

It could be questioned whether under the present experimental conditions HR animals were really cumulatively exposed to more corticosterone than LR mice. This seems very likely, although basal plasma corticosteroid levels were not statistically different between the experimental groups. The latter could be partly explained by the fact that the current measurements were single time-point observations, which are liable to considerable variation due to conditions in the animal house that cannot be entirely controlled for. We cannot exclude that the two-fold difference in averaged basal corticosteroid levels between HR and LR mice might become statistically meaningful with multiple sampling or at a later age. This is supported by results from previous studies in HR compared to IR and LR mice, monitoring the circadian glucocorticoid secretion pattern. These studies revealed clearly increased trough corticosterone levels in HR mice, resulting in a flattened diurnal rhythm of HPA axis activity [27], [29]. Such differences in circulating corticosteroid levels may become apparent at other moments of the circadian cycle than were presently tested. Importantly, though, there was a clear and significant difference in stress-induced levels of corticosterone between the three experimental groups. Thus it is very likely that daily stressors – both internal (social stressors e.g. hierarchical differences) and external (e.g. being transferred from one cage to another as part of the animal care procedure or unpredicted noises in the animal facility) – had increasing impacts on the HPA axis activity of animals from low to high stress reactivity, respectively. It is a limitation of the current experimental design, though, that an exact quantification of this increase in exposure cannot be provided.

It is remarkable that, despite the clear differences in stress reponsivity between the three mouse lines, as explained above, gross morphological changes were not present in any of the limbic brain regions. In this study we focused on adolescent mice (∼7 weeks at decapitation), which is relevant when one is clinically interested in the premorbid differences associated with psychiatric disorders [56]–[60]. While we observed no marked differences in dendritic complexity under the current non-stressful experimental conditions, we cannot exclude that differences may become apparent when animals are studied shortly after acute stress exposure. Earlier, HR compared to LR mice (albeit a few weeks older) were found to be hyperactive, showed more explorative behavior, and struggled more in the tail-suspension test as well as the forced swim test [27], pointing to a distinct endophenotype with respect to hippocampus- and amygdala-dependent behavioral parameters under relatively stressful conditions. This supports that the relevance of genetic differences for behavioral responses may indeed be more apparent in combination with rather stressful test conditions in adulthood (such as the tail-suspension test and forced swim stress). Yet, deficits in HR hippocampal function were also clearly visible in tests that are not very stressful, such as the Y-maze and an object recognition tasks [28].

The consequences of differences in stress reactivity may also amplify with age. Thus, the impact of stressful experiences on a juvenile brain, where the HPA axis is still developing, can lead to different effects when compared to the adult brain [61], [62]. For instance, juvenile stress was reported to decrease both fear and anxiety-like behaviors when tested at adolescence but not in adulthood [63] and to be associated with a reduction in cFos-positive cells in the amygdala during adolescence compared to adulthood [64]. Moreover, exposure to juvenile stress was found to increase emotionality and depression-like symptoms following a subsequent stress episode in adulthood [65], [66].

Earlier studies have shown that chronic stress in adulthood causes a reduction in the spine density of CA1 pyramidal cell apical dendrites, while these conditions are generally insufficient to change dendritic complexity in CA1 neurons [50], [51]. Spine density thus seems a sensitive measure of cumulative corticosterone exposure. Interestingly, based on group averages, we found that spine density of distal apical tertiary branches of CA1 neurons was significantly higher in cells from LR compared to (IR and) HR animals. The positive finding with respect to spine density emphasizes that our model was not too subtle to observe any morphological changes at all. We did not elaborate possible differences at this finer level of detail between the three lines in other areas, since this was not the primary goal of our study. The higher spine density in tertiary branches of CA1 neurons from LR compared to HR animals and the somewhat larger basal dendrites of CA1 neurons in the LR group suggest that the presumably low cumulative corticosterone exposure of these animals may protect them to some extent against dendritic remodeling in the light of daily stressors.

In conclusion, mice with a genetic predisposition to stress hyper-reactivity and presumably higher cumulative life-exposure to corticosterone show, at least in late adolescence, a remarkable resilience with respect to the dendritic morphology of principal neurons in limbic areas which play a key role in mood and anxiety disorders. If such resilience would also exist in the human brain, genetic predisposition per se may not be sufficient to cause substantial structural plasticity. This could explain why volume changes associated with psychopathology (e.g. in major depression) are usually very small [53], [67] and only prominent in combination with strong adversity particularly during early life [68], [69]. Spine density, however, seems more plastic and may contribute to cognitive dysfunction even in the absence of gross morphological changes.
